# Vaginal progesterone for prevention of preterm birth in asymptomatic high-risk women with a normal cervical length: a systematic review and meta-analysis protocol

**DOI:** 10.1186/s13643-021-01702-9

**Published:** 2021-05-21

**Authors:** Kimberley P. Williams, Liam McAuliffe, Rosanna Diacci, Anne-Marie Aubin, Ashad Issah, Carol Wang, Jason Phung, Craig E. Pennell

**Affiliations:** 1 School of Medicine and Public Health, The University of Newcastle, Callaghan, New South Wales Australia; 2 Mothers and Babies Research Centre, Hunter Medical Research Institute, New Lambton Heights, New South Wales Australia; 3 Maternity and Gynaecology, John Hunter Hospital, New Lambton Heights, New South Wales Australia

**Keywords:** Progesterone, Preterm birth, Cervical

## Abstract

**Background:**

Preterm birth (PTB) is estimated to affect 14.9 million babies globally every year. Global rates of PTB continue to increase from 9.8 to 10.6% over a 15-year period from 2000 to 2014. Vaginal progesterone is commonly used by clinicians as a prevention strategy, with recent evidence affirming the benefit of vaginal (micronised) progesterone to prevent PTB in women with a shortened cervix (< 25 mm). Given the low incidence of a short cervix at mid-gestation in high-risk populations further evidence is required. The objective of this review is to determine if vaginal progesterone reduces spontaneous preterm birth (sPTB) before 37 weeks in asymptomatic high-risk women with a singleton pregnancy with a normal mid-gestation cervical length.

**Methods:**

Studies will be sourced from MEDLINE, Embase and Cochrane Register of Trials (CENTRAL) from their inception onwards with the search terms progesterone and preterm birth. Studies will be screened and included if they assess vaginal progesterone compared to placebo in women with a normal cervical length. The primary outcome will be sPTB < 37 weeks, with secondary outcomes of sPTB < 34 weeks.

Two independent reviewers will conduct study screening at abstract and full text level, data extraction and risk of bias assessment with disagreements resolved by an experienced researcher. The Mantel-Haenszel statistical method and random effects analysis model will be used to produce treatment effect odds ratios and corresponding 95% confidence intervals.

**Discussion:**

This review will assess the current body of evidence and provide clarity regarding the potential benefits and best practice of use of vaginal progesterone in asymptomatic women with high-risk singleton pregnancies and normal cervical length.

**Trial registration:**

PROSPERO CRD42020152051

**Supplementary Information:**

The online version contains supplementary material available at 10.1186/s13643-021-01702-9.

## Introduction

Preterm birth (PTB) is estimated to affect 14.9 million babies worldwide every year [1], with a higher burden in low- and middle-income countries [2]. This has generated a geographic divide with several European countries such as Finland and Sweden having the lowest PTB rates < 6%, whilst low income countries such as Malawi in Africa, have the highest PTB rate of 18.1% [3]. Similarly, estimated global PTB rates have increased from 9.8% in 2000 to 10.6% in 2014 [2]. Despite approximately 80% of these preterm births occurring in disadvantaged countries in sub-Saharan Africa and Asia, data suggests that rates of PTB are also steadily increasing in high-income countries [2]. This is a worrying trend as PTB remains the leading cause of death for children under the age of five [4]. Furthermore, it increases the risk of short-term complications including infant respiratory distress syndrome and intraventricular haemorrhage, as well as life-long impacts including increased risk of neurodevelopmental delay and adult non-communicable diseases [5].

A number of interventions have been shown to significantly decrease the rate of spontaneous preterm birth (sPTB) including specialist antenatal clinics for women at high risk of PTB [6]; cervical cerclage for women with a previous sPTB and/or a short cervix (< 25 mm) [7, 8] and vaginal progesterone for women with cervical length < 25 mm [9,13]. Progesterone decreases uterine contractility through inhibition of production of prostaglandins in the myometrium [14] and has been shown to be important in maintaining a pregnancy until term [15].

A recent individual patient data (IPD) meta-analysis affirmed the benefit of vaginal progesterone in prevention of sPTB in women with a short cervix (< 25 mm) with a 20% reduction in sPTB before 36 weeks, and a greater reduction of 35% before 34 weeks [8]. This IPD suggested that vaginal progesterone is still beneficial even in nulliparous women with only a short cervix as a risk factor [8]. As only 5.8-7.3% of high-risk women [9, 10], and 0.45-1.68% in unselected populations [11,13] have a short cervix at mid gestation, the number of women who benefit from progesterone is relatively low. It is currently unclear whether vaginal progesterone would be of benefit to the other high-risk women with a normal mid-gestation cervical length (> 25 mm).

Whilst there is a substantial body of research into progesterone and PTB prevention, current literature focuses on women with a short cervix and fails to address whether vaginal progesterone would benefit high-risk women specifically with a normal mid-gestation cervical length (> 25 mm). Many studies assessing the effectiveness of vaginal progesterone in based on risk factors alone did not concurrently measure the cervical length and therefore we cannot be confident that these populations did not contain patients with a short cervix [16]. The most recent systematic review assessing the role of progesterone in PTB reduction [17] has done so by combining studies where progesterone was administered orally, vaginally and intramuscularly (17-OHP). There is evidence to suggest that the mechanism of action differ [18] and therefore should be studied separately. This is supported by the fact that vaginal progesterone and IM 17-OHP appear to be effective in different populations [19, 20]. Whilst several publications comparing the route of administration exist [21], this is beyond the scope of this review.

Recommendations currently exist for the administration of vaginal progesterone for women with a short cervix and therefore many centres in high resource countries are able to offer cervical length screening as routine care. We know from prior publications that the rate of a short sonographic cervix is low [10]. Therefore, this proposed systematic review will answer the question does vaginal progesterone reduce spontaneous PTB in asymptomatic high-risk women with a singleton pregnancy with a normal mid-gestation cervical length?

## Methods

The present protocol has been registered within the PROSPERO database (registration number CRD42020152051) and is being reported in accordance with the reporting guidance provided in the Preferred Reporting Items for Systematic Reviews and Meta-Analyses Protocols (PRISMA-P) statement (see checklist in Additional file 1).

### Information sources and search strategy

The search strategy will be developed focusing on identifying the relevant intervention with no population-related keywords. The primary source of literature will be a structured search of the electronic databases MEDLINE, EMBASE and Cochrane Register of Trials (CENTRAL) from their date of inception onwards. The secondary source of literature will be a search of the grey literature, including Google Scholar. We will perform hand-searching of the reference lists of included studies, relevant reviews, national clinical practice guidelines or other relevant documents. We will contact content experts and authors who are prolific in the field. The literature searches will be designed and conducted by the review team with an experienced health information specialist. The search will include the following search terms: preterm birth OR premature birth AND progesterone. We will place no restriction on the length of study follow-up time, or on country, year or language of publication. However, the search will be limited to studies on humans. Medical subject headings (MeSH) will be used when relevant. Where appropriate, the authors of studies will be contacted to provide additional information. A draft search strategy for MEDLINE is provided in Additional file 2.

### Eligibility criteria

The eligibility of studies included will be based on inclusion and exclusion criteria applied to the domains of participant, exposure, comparator, study type and outcome.

#### Participants

This review will consider all studies that include asymptomatic pregnant women being treated with vaginal progesterone. We will exclude studies that include women with a short cervix or who were symptomatic. Studies that exclusively studied women with multi-gestation will be excluded.

#### Intervention and comparators

Studies comparing vaginal progesterone either compared to placebo or no treatment will be included. Studies that involved other methods of progesterone administration such as intramuscular injection will be excluded.

Vaginal progesterone is available as a gel, suppository or pessary [14]. It is the most bioavailable form of progesterone for uterine and cervical effects with the fewest side effects. Its micronisation decreases particle size and increases surface area. This results in improved absorption with less metabolic and vascular side effects [22]. The vaginal route also allows rapid absorption and avoids first pass hepatic metabolism, resulting in high bioavailability in the uterus [23].

#### Outcomes

The primary outcome is sPTB before < 37 weeks gestation.

Preterm birth will be defined as live or stillbirth with a gestational age between 20 and 37 weeks.

The secondary outcome is sPTB before < 34 weeks gestation.

#### Types of studies

The review will include randomised controlled trials. All included papers must include vaginal progesterone compared to either placebo or no treatment. Those studies which also present a control group will be included.

We will place no restriction on the country, year and language of publication or the length of study follow-up. Included studies will be limited to human trials.

### Selection and data collection process

#### Study selection

Titles and abstracts identified through all sources will be downloaded to Endnote [24] and duplicates will be removed. Studies will then be screened using the specified eligibility criteria above and studies that do not meet the criteria will be excluded. Full texts of remaining studies will be screened before undergoing critical appraisal and data extraction. All levels of screening will be conducted by two independent reviewers and any disputes between reviewers will be resolved by an independent moderator. None of these reviewers will be blinded to titles, authors, journals or institutions.

#### Data management

The search will be uploaded to an Endnote [24] library, which allows collaboration between multiple reviewers during the study selection process.

#### Data collection

Two reviewers will extract data through Endnote [24] using a standardised electronic data extraction sheet. Any discrepancies will be moderated by a third senior research reviewer. Once extracted, upon reviewer agreement, data will be transferred into Review Manager version 5.3 data-analysis software [25].

The following data will be extracted:

- Study characteristics: authors; publication date; study design; country of study; sample size; confounding factors of participants; publication status; trial size; funding and risk of bias information.
- Intervention characteristics: type of intervention used; reason for intervention; patient characteristics (maternal age, gravity, parity, cervical length) and any co-interventions received.
- Outcomes: maternal, foetal and neonatal outcome data and definitions of each of the outcomes as described below.

#### Risk of bias in individual studies

Risk of bias for each paper will be assessed using the Joanna Briggs Institute critical appraisal method for randomised control trials [26]. Studies which are deemed to have not addressed the possibility of bias in the design, conduct or analysis will then be excluded.

#### Confidence of cumulative evidence

To assess the strength of the body of evidence, we will utilise the Cochrane GRADE tool [27]. Evidence will be assessed in terms of risk of bias, consistency, directness, precision and publication bias. With regard to GRADE, quality will be assessed as being one of 4 grades: (i) highwe are very confident that the true effect is close to that of the estimate of the effect; (ii) moderatewe are moderately confident in the effect estimate, and the true effect is likely to be close to the estimate of the effect, but there is a possibility that it is substantially different; (iii) lowour confidence in the effect estimate is limited, and the true effect may be substantially different from the estimate of the effect; and (iv) very lowwe have very little confidence in the effect estimate, and the true effect is likely to be substantially different from the estimate of effect. Two independent reviewers will conduct the assessment, with discrepancies resolved through discussion and consensus between the two reviewers, or consultation with a third reviewer.

Review Manager version 5.3 [25] will be used to compute graphic representations of potential bias within and across studies.

### Data synthesis

Meta-analysis will be constructed by pooling studies using Covidence and RevMan 5.3. In order for synthesis, studies must report outcomes appropriately (i.e. < 37 for the primary outcome) or provide sufficient information to calculate the outcome(s) of interest. We will synthesise published data from the original manuscripts, appendices or any data provided by authors upon request. We will only synthesise data based on the primary research question of vaginal progesterone vs. placebo and therefore will accept varying doses provided they have the correct route of administration. Given the broad nature of the at risk population, we expect there to be significant heterogeneity within these studies and will test for this using statistical methods such as Chi^2^ and I^2^ statistics.

If feasible and appropriate, outcome data will be used to perform random effects meta-analyses because of anticipated heterogeneity. The random effects model assumes the study level effect estimates follow a normal distribution, considering both within-study and between-study variation.

Dichotomous outcomes will be assessed and reported using risk ratios and 95% confidence intervals will be used. For continuous outcomes, trial data will be reported using mean differences (MDs) or standardised mean differences (SMDs) and 95% continuous outcome variables. Where meta-analysis is not possible, alternative synthesis methods, including summarising effect estimates, will be used as recommended by the Cochrane Handbook for Systematic Reviews of Interventions [28].

#### Missing data

For studies which present missing data, we will attempt to contact authors. However, if this is not possible, we will conduct sensitivity analysis which will exclude trials with > 30% missing data.

#### Meta-bias(es)

Meta-biases will be assessed by examining funnel plots for symmetry and Eggers test may be used for assessing publication bias.

#### Additional analyses

Sensitivity analysis will be conducted on the primary outcome for sPTB < 37weeks gestation by removing studies which are judged to have an overall high risk of bias, allowing us to examine their impact on the effect estimate of the primary outcome. Data will be extracted from each individual study regarding baseline maternal characteristics (age, gestation, risk of bias, setting and geographical region) and neonatal outcomes where individual patient data is available and pertinent sub-group analyses will be conducted if there are vast differences in baseline characteristics.

## Discussion

This systematic review and meta-analysis aims to determine the effectiveness of vaginal progesterone for prevention of PTB in asymptomatic high-risk women with a normal cervical length. It is hoped this paper will synthesise multiple recent large scale trials to provide valuable therapeutic information to specialists in their clinical decisions for women at risk of PTB. It is hoped women at high risk of obstetric complications, their families and the wider community will benefit from these findings. The results of this paper will help to inform guidelines and reduce the short- and long-term negative health outcomes of preterm birth.

Whilst we do not anticipate any major operational issues in the conduction of this review, an issue we foresee as possible would be if the included studies do not have individual patient data. If this is the case, we plan to overcome this by contacting the authors of the individual studies about whether individual patient data is available.

Firstly, in terms of study selection, there may not be enough studies in the area to yield significant results and/or these studies may be of poor quality. Secondly, studies may not report individual patient data and may only include summary of results which limits the analyses that can be reached in our meta-analysis.

Should there be any major changes that the existing protocol that significantly affect the scope of the investigation, safety of the subjects or quality of the study, a protocol amendment will be submitted.

### Plan for dissemination

We plan for this systematic review and meta-analysis to be disseminated through peer-reviewed publication.

## Supplementary Information

**Additional file 1.** PRISMA-P Checklist

**Additional file 2.** Search Strategy

## Data Availability

Not applicable.

## Abbreviations

CI: Confidence interval
CINAHL: Cumulative Index of Nursing and Allied Health Literature
GRADE: Grading of Recommendations, Assessment, Development and Evaluations
IPD: Individual patient data
MD: Mean difference
MeSH: Medical subject headings
P value: Probability value
PRISMA-P: Preferred Reporting Items or Systematic Reviews and Meta-Analyses Protocol
PPROM: Preterm premature rupture of membranes
PROSPERO: International Prospective Register of Systematic Reviews
RCT: Randomised control trial
RDS: Respiratory distress syndrome
RevMan: Review Manager 5.3
ROBINS I: Risk of Bias in Non-Randomised Studies of Interventions
ROBINS II: Risk of Bias in Randomised Studies of Interventions
RR: Risk ratio
SPTB: Spontaneous preterm birth
SMD: Standardised mean difference
