# Supplementary material for: Vaginal progesterone for prevention of preterm birth in asymptomatic high-risk women with a normal cervical length: a systematic review and meta-analysis protocol
Source: Syst Rev. 2021 May 21;10:152. doi: 10.1186/s13643-021-01702-9 (PMC8139044; doi:10.1186/s13643-021-01702-9)
Supplement: Supplementary file 2 — Additional file 2. Search Strategy [file 13643_2021_1702_MOESM2_ESM.docx]

**Additional File Two – Search Strategy**

1. Preterm OR Premature

2. Birth

3. 1 AND 2

4. Progesterone

5. 3 AND 4
